# Children on the Gaza-Israel Border: Victims of War

**DOI:** 10.3389/phrs.2024.1607192

**Published:** 2024-04-24

**Authors:** Ora Paltiel, Orly Manor, Ronit Calderon Margalit, Orna Baron Epel, Yael Bar Zeev, Elliot Berry, A. Mark Clarfield, Eldad J. Dann, Nadav Davidovitch, Milka Donchin, Manfred Green, Hagit Hochner, Yehuda Neumark, Dorit Nitzan, Ari Paltiel, Oliver Razum, Bruce Rosen, Mary Rudolf

**Affiliations:** ^1^ Hadassah-Hebrew University Braun School of Public Health and Community Medicine, Faculty of Medicine, Hebrew University of Jerusalem, Jerusalem, Israel; ^2^ School of Public Health, Faculty of Social Welfare and Health Sciences, University of Haifa, Mount Carmel, Israel; ^3^ Centre of Global Health, Faculty of Health Sciences, Ben-Gurion University of the Negev, Beer-Sheva, Israel; ^4^ The Ruth and Bruce Rappaport Faculty of Medicine, Technion Israel Institute of Technology, Haifa, Israel; ^5^ Department of Health Policy and Management, School of Public Health, Ben Gurion University of the Negev, Beer-Sheva, Israel; ^6^ School of Public Health, Faculty of Health Sciences, Ben-Gurion University of the Negev, Beer-Sheva, Israel; ^7^ Center for the Study of Preparedness and Response to Emergencies and Disaster, Faculty of Health Sciences, Ben-Gurion University of the Negev, Beer-Sheva, Israel; ^8^ Department of Epidemiology and International Public Health, School of Public Health, Bielefeld University, Bielefeld, Germany; ^9^ Paul Baerwald School of Social Work and Social Welfare, Hebrew University of Jerusalem, Jerusalem, Israel; ^10^ Population Health, Azrieli Faculty of Medicine, Bar Ilan University, Safed, Israel

**Keywords:** humanitarian crises, child and maternal malnutrition, conflict-affected populations, post traumatic stress disorder, child development

In Israel and Gaza, following the atrocities of 7 October 2023 and the ensuing war, children cannot fail to wonder, “Am I next?”. Adults are at a loss for reassuring words.

Armed conflict damages the physical and mental health of children. War disrupts the social determinants of health as well as the healthcare systems designed to maintain it. The economic instability, damaged living conditions, and limited access to essential services caused by war lead to malnutrition, food insecurity and desperation, all of which increase susceptibility to disease. Displacement and violence expose children to physical and psychological trauma, with potential life-long consequences. Disruptions in learning further jeopardize their wellbeing and development. Fear of the other leads to dehumanization.

All this is well known. Humanitarian efforts seek to limit harm to children, but they require international cooperation, which itself is hampered by conflict. Disruptions in the delivery of multifaceted aid exacerbates wartime crises. The current situation in Gaza and Israel presents specific challenges and portends long-term physical, emotional, and developmental damage to a generation of children.

International media are filled daily with reports of deaths, maiming and suffering of Gaza’s children. Hundreds of thousands have been displaced, and have lost their homes. Gaza’s population is extremely young, with nearly half [1] the population under the age of 18 years; thus, when civilians are inadvertently injured during military activity, or deliberately and cynically used as human shields [2], a large proportion of the victims are expected to be children.

Furthermore, when an entire population experiences food insecurity, the most insecure and vulnerable will be children, women, older and chronically ill persons. The nutritional status of Gazan children and pregnant/lactating mothers was already poor by November 2023 [3], with an estimated half a million requiring preventive or curative nutritional interventions. Childhood wasting and stunting were already common, as was anemia in women. The first 1,000 days [4] are critical for child development, both physically and mentally, and damage can be irreversible. Moreover, poor nutrition in pregnancy can contribute to adverse metabolic, cardiovascular and mental health outcomes in their offspring, even into adulthood [5]. UNICEF has reported that food intake among Gazans, especially children, has been severely restricted, with up to one in four enduring famine-like starvation [6]. Malnutrition is compounded by widespread outbreaks of diarrhea.

Unfortunately, as the conflict continues, living standards in Gaza will only worsen. Agriculture has been halted and much civilian infrastructure including schools and health facilities have been damaged. When supplies do arrive, children are the least able to fight for their own survival and obtain them. Food aid is not equitably distributed, and may be looted or commandeered by Hamas or related organizations [7].

The government and people of Israel must be attentive to the plight of children in Gaza, since the long-term consequences of ignoring their hunger, displacement and fears will only result in a vicious cycle of further brutalization, radicalization and hatred, which will fuel the conflict for years to come. As an outlet for their ambitions and sense of purpose in such inhumane circumstances, or merely to avoid hunger and unemployment, children might opt or be forced to join Hamas or other terrorist organizations, a “reasonable” option, especially if they have been indoctrinated to hate.

It is imperative that humanitarian aid, especially food, water, medications and vaccines be allowed to enter Gaza in adequate quantities, and that international aid organizations guarantee that this aid reaches its legitimate target, the children of Gaza. Not an easy task, but a necessary one. Israel, Egypt and their international governmental and nongovernmental partners need to further augment and improve the scale and coordination of aid and rehabilitation efforts. It is understandable that some Israelis, especially those with relatives still held captive in Gaza, are reluctant to enable the transfer of aid *via* Israel, having received no signs of life or assurances of the delivery of essential medicines from their loved ones. The Israeli nation, after experiencing an unprecedented national trauma on 7th October, and ongoing grief thereafter, struggles to express empathy for Gazans. As eloquently stated by Yuval Noah Harari, “*The mind is filled to the brim with our own pain, and no space is left to even acknowledge the pain of the others*.” [8] However, the government must rise above this anguish and “do the right thing” from an ethical, legal and humanitarian point of view, and not least in order to protect the interests of Israel on the world stage.

Tragically, international media pay far less attention to the fact that on the Israeli side of the border, children have been subjected to thousands of mortar and rocket attacks since 2007. Some commentators have bizarrely termed this situation “normal”. Not surprisingly, this ongoing threat has caused widespread PTSD [9], greatly aggravated since the pogrom of 7 October. Tens of thousands have been displaced from their homes and schools. Acutely, as a direct result of the Hamas assault, scores of children were orphaned, some of both parents. Many were abducted from their homes after witnessing horrific violence, incarcerated above and underground in atrocious conditions. They, like their Palestinian counterparts, experienced hunger, malnutrition and disease. A baby and toddler, kidnapped with their mother, remain hostages. The 7 October events, the subsequent war and continuing rocket attacks on Jews and Arabs have impacted mental health of children all over Israel. Most know every detail of these horrors and not surprisingly many are frightened and devastated by this knowledge. Adults struggle to comfort them. High levels of stress, anxiety and fear were detected in a sample of Israeli children in mid-November [10]. Traumatized by the current conflict and from ongoing threats of Hamas attacks, these children will continue to be fearful of their Palestinian neighbours—hardly a recipe for coexistence.

While addressing the needs of Gaza, international bodies must pressure Hamas to release all hostages and desist immediately from firing rockets indiscriminately at civilian targets throughout Israel. Just as urgently, they must condemn Hezbollah for recklessly endangering civilians on both sides of the Lebanon-Israel border where tens of thousands of children have been displaced from their homes and traumatized by constant shelling from this terrorist group.

The situation of the children in Gaza is acute, but we must remember, there are children on both sides of this war-torn border. As experts in public health, our professional knowledge and experiences fill us with fears for the future, especially that of the children. Preserving their health and safety is a security issue for the entire region.
